# Supplementary material for: The interactome of intact mitochondria by cross-linking mass spectrometry provides evidence for coexisting respiratory supercomplexes
Source: Mol Cell Proteomics. 2017 Dec 8;17(2):216–32. doi: 10.1074/mcp.RA117.000470 (PMC5795388; doi:10.1074/mcp.RA117.000470)
Supplement: Supplemental Data [file supp_RA117.000470_133922_0_supp_23978_fzffwf.zip › spectra_annotation/mito_spectra_annotation/480-2-11-1-13-1.pdf]

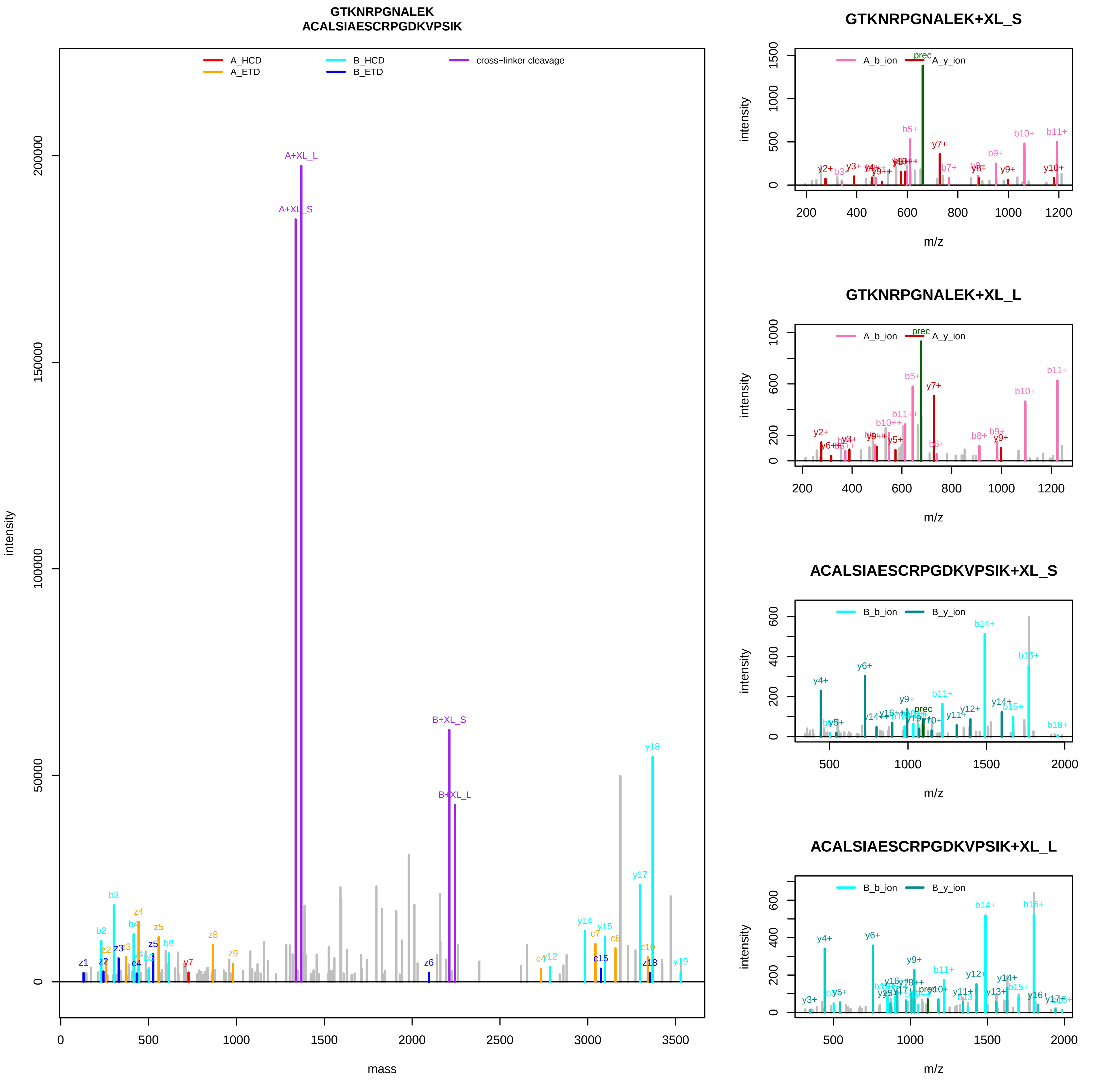

GTKNRPGNALEK
ACALSIAESCRPGDKVPSIK
A_HCD
A_ETD
B_HCD
B_ETD
cross−linker cleavage
A+XL_L
A+XL_S
B+XL_S
B+XL_L
b2
b3
b4
b5
b6
z1
z2
z3
z4
z5
z6
z8
z9
c4
c7
c8
c10
c15
y7
y12
y14
y15
y17
y18
y19
z18
intensity
mass
0
50000
100000
150000
200000
0
500
1000
1500
2000
2500
3000
3500
GTKNRPGNALEK+XL_S
A_b_ion
A_y_ion
prec
b5+
b10+
b11+
y7+
b9+
y10+
intensity
m/z
0
500
1000
1500
200
400
600
800
1000
1200
GTKNRPGNALEK+XL_L
A_b_ion
A_y_ion
prec
b5+
y7+
b10+
b11+
b8+
b9+
y9+
y2+
intensity
m/z
0
200
600
1000
200
400
600
800
1000
1200
ACALSIAESCRPGDKVPSIK+XL_S
B_b_ion
B_y_ion
b14+
b16+
y4+
y6+
y9+
b11+
y12+
y14+
b15+
b18+
prec
intensity
m/z
0
200
400
600
500
1000
1500
2000
ACALSIAESCRPGDKVPSIK+XL_L
B_b_ion
B_y_ion
b14+
b16+
y4+
y6+
y9+
b11+
y12+
y14+
b15+
y3+
prec
intensity
m/z
0
200
400
600
500
1000
1500
2000
